# Figure-ground segmentation based on motion in the archerfish

**DOI:** 10.1007/s10071-024-01873-7

**Published:** 2024-04-15

**Authors:** Svetlana Volotsky, Ronen Segev

**Affiliations:** 1 Department of Biomedical Engineering, Ben-Gurion University of the Negev, Beersheba, Israel; 2 School of Brain Sciences and Cognition, Ben-Gurion University of the Negev, Beersheba, Israel; 3 Department of Life Sciences, Ben-Gurion University of the Negev, Beersheba, Israel

**Keywords:** Archerfish, Visual object recognition, Segmentation, Figure ground

## Abstract

Figure-ground segmentation is a fundamental process in visual perception that involves separating visual stimuli into distinct meaningful objects and their surrounding context, thus allowing the brain to interpret and understand complex visual scenes. Mammals exhibit varying figure-ground segmentation capabilities, ranging from primates that can perform well on figure-ground segmentation tasks to rodents that perform poorly. To explore figure-ground segmentation capabilities in teleost fish, we studied how the archerfish, an expert visual hunter, performs figure-ground segmentation. We trained archerfish to discriminate foreground objects from the background, where the figures were defined by motion as well as by discontinuities in intensity and texture. Specifically, the figures were defined by grating, naturalistic texture, and random noise moving in counterphase with the background. The archerfish performed the task well and could distinguish between all three types of figures and grounds. Their performance was comparable to that of primates and outperformed rodents. These findings suggest the existence of a complex visual process in the archerfish visual system that enables the delineation of figures as distinct from backgrounds, and provide insights into object recognition in this animal.

## Introduction

Many animals’ visual interaction with the world relies heavily on the ability to detect and recognize objects in the environment. It is believed that surface-segmentation, i.e., the ability to delineate an object from the surrounding background, is one of the first critical stages in visual scene analysis. Thus, to better understand the computational basis of vision in different animals, more must be known about how this information processing component performs tasks.

**Fig. 1:**
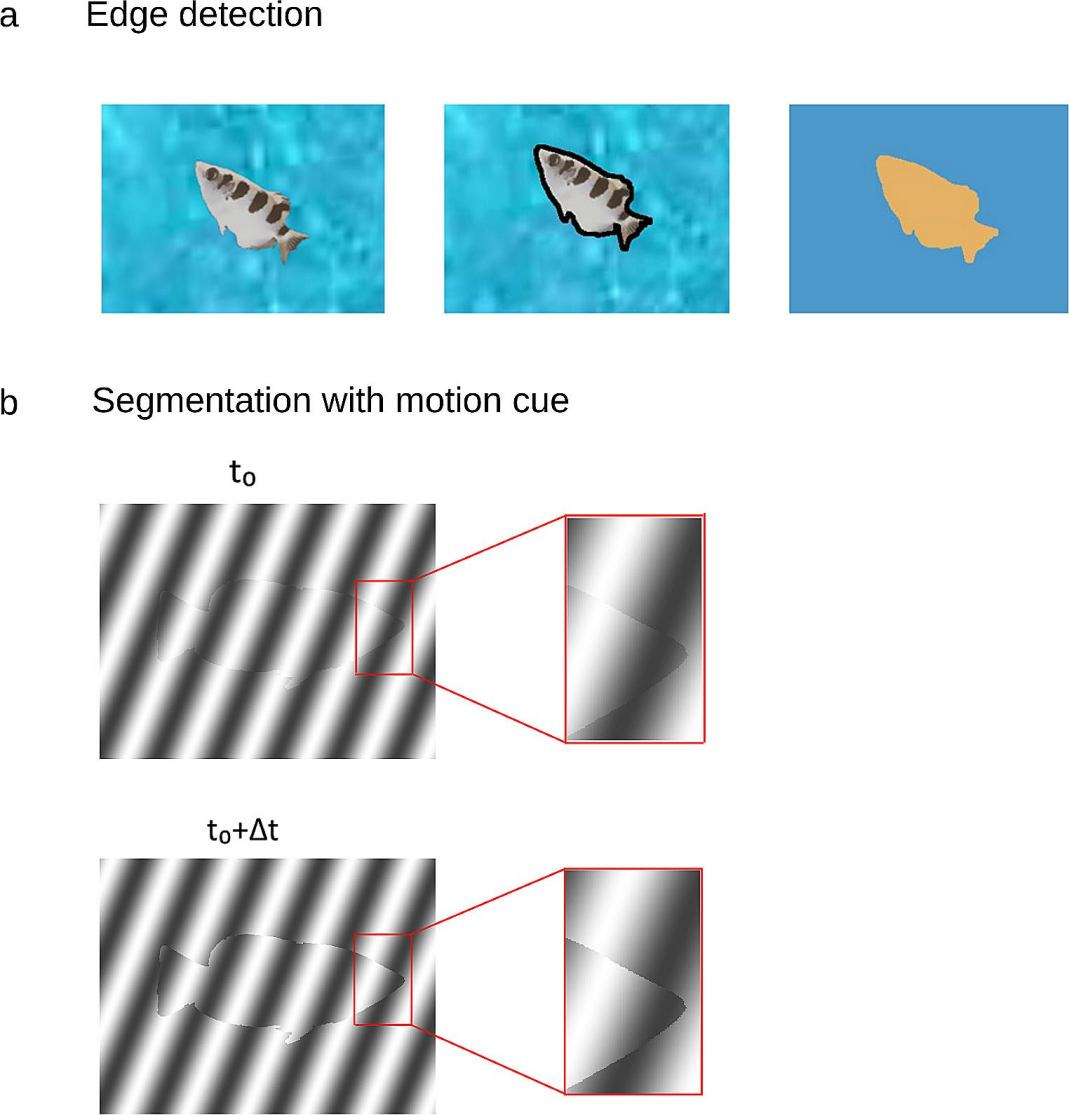
Segmentation is a crucial step in visual scene processing and object recognition. **(A)** Figure-ground segmentation can be performed by edge detection based on discontinuity of intensity or color. **(B)** Motion cues can be used to detect the boundary of the foreground by deletion-accretion of pixels due to the motion of the foreground over the background. By comparing the deletion and appearance of pixels resulting from object motion, the foreground can be segmented from the background

The key visual features that identify the border of an object or surface are the discontinuities or abrupt changes at the figure border (Lamme 1995; Lamme and Roelfsema 2000; Zhou et al. 2000); i.e., changes in the properties of the surface such as luminance, orientation, or texture (Fig. 1a). However, there are also discontinuities in these visual features in the interior of objects or surfaces. This makes the detection of actual borders using ambiguous cues a challenge to any visual system.

Two cues signaling object borders are however unambiguous; namely, accretion and deletion, which are constituted by the appearance or disappearance of pixels forming the background surface due to motion of the foreground object (Fig. 1b; (Lamme 1995; Stoner and Albright 1996). These are the only cues that remain unambiguous, invariant to texture, and they do not require explicit object recognition. These cues exist locally and do not require global processing of the visual scene (Gibson 2014; Tsao and Tsao 2022).

Behaviorally, primates’ ability to detect object borders based on accretion and deletion has been tested in several species and differs considerably from that of rodents (Luongo et al. 2023; Schnabel et al. 2018). Several primate species can perform segmentation based on motion signal, both when the stimulus was based on grating, when local orientation information was present, in cases where the figure and ground were derived from naturalistic noise images, and where there was no orientation information present (Ho et al. 2021; Luongo et al. 2023; Mustafar et al. 2018).

Several rodent species were found to have limited capacity to perform surface segmentation based on accretion and deletion alone. Rodents were able to perform the figure-ground segmentation task when local orientation information could be used to segment the figure from the ground but failed to do so when the stimulus was derived from naturalistic noise images where no orientation information could be tapped (Luongo et al. 2023).

These differences between primates and rodents indicate that the visual systems of different lineages perform very differently on the surface segmentation task which is so central to our understanding of visual scene processing. Clearly, to better grasp whether and how surface segmentation is performed across vertebrates’ visual systems, other species should be tested.

This article reports on a teleost fish’s capacity to segment the foreground surface from the background surface. We capitalized on our ability to perform controlled experiments in the archerfish (*Toxotes Chatareus*). This fish is known for its ability to shoot insects above the water level for food (Ben-Tov et al. 2018; Lüling 1963; Newport and Schuster 2020; Ben-Simon et al. 2012). The archerfish can be trained to shoot at targets on a computer monitor, which enables controlled experiments. In recent years, studies have shown that the archerfish can recognize natural (Volotsky et al. 2022; Newport et al. 2018) and artificial (Newport et al. 2014, 2015) objects. In addition, recording of single cells in the early visual system of the archerfish (Ben-Tov et al. 2013; Reichenthal et al. 2018) showed that it has characteristics similar to those found in the early visual system of mammals, and primates in particular. This includes tuning to orientation of surfaces and contextual modulations which can be used for early processing in segmenting (Ben-Tov et al. 2013, 2015). Thus, the archerfish provides an excellent opportunity to study the mechanisms of object recognition in fish.

As described below, archerfish were administered several segmentation tasks involving different background textures with and without a motion cue. The results showed that the archerfish can perform segmentation tasks that are purely defined by motion, i.e., the accretion and deletion of pixels. In addition, the archerfish successfully discriminated foreground objects from the background with a static target based on the discontinuity of texture. We compare these archerfish results to several mammalian species to better understand this behavior across taxa.

## Methods

### Animals

Eight archerfish were used in the experiments. Adult fish (6–14 cm in length; 10–18 g) were purchased from a local supplier. Throughout the experiments, the fish were kept separately in 100-liter aquaria filled with brackish water at 25^o^-29^o^C on a 12–12 h light-dark cycle. Fish care and experimental procedures were approved by the Ben-Gurion University of the Negev Institutional Animal Care and Use Committee and were in accordance with government regulations of the State of Israel.

### Training

**Fig. 2:**
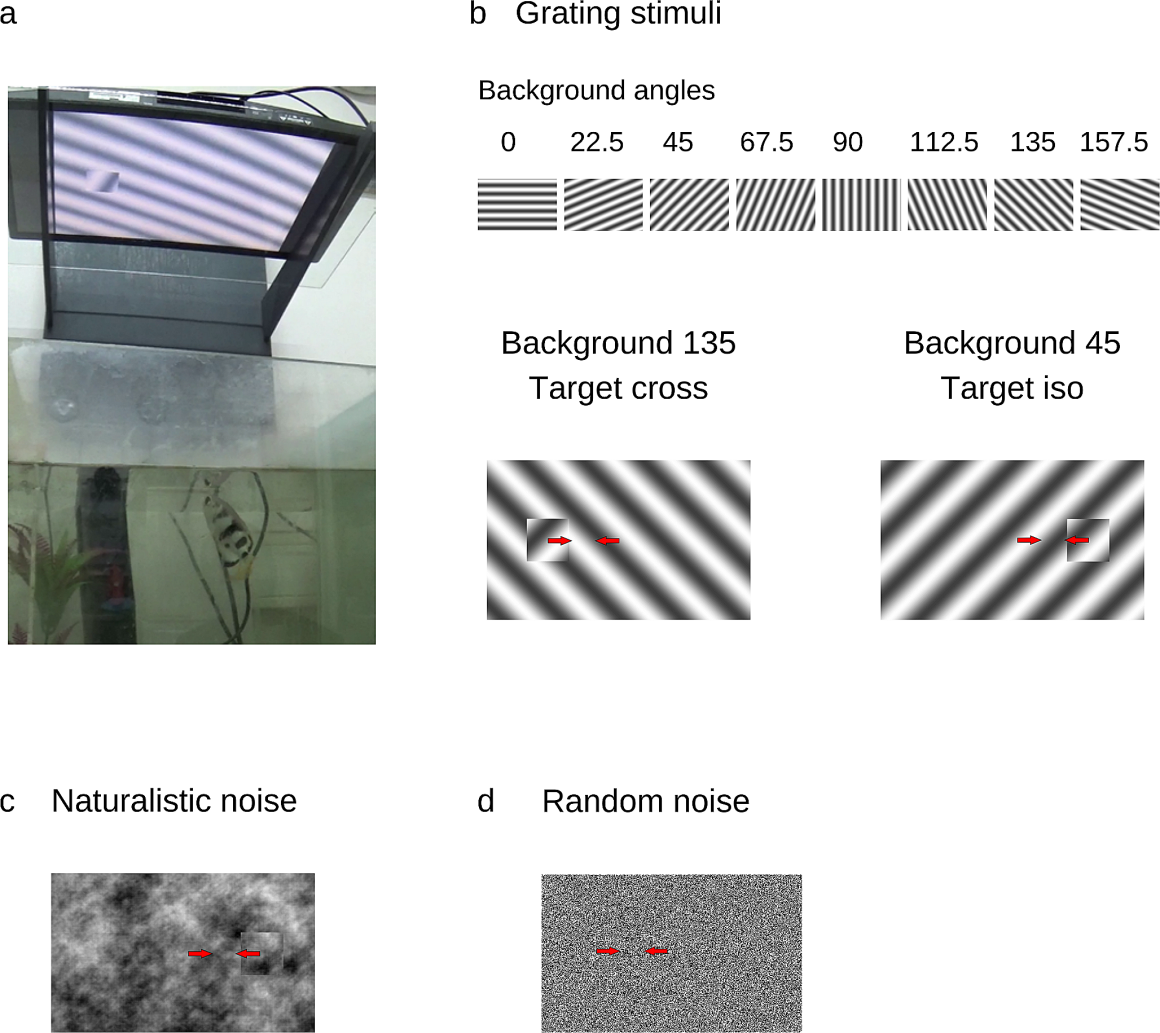
Experimental setup and different stimuli. **(A)** Example of archerfish during the behavioral experiments. A screen is placed above water level and the stimulus is presented. If the fish shoots at the target, it is rewarded by placing a food pellet in the water. **(B)** Grating experiment: the background orientation is selected from eight different orientations. The target is oriented either 90^o^ from the background (Cross condition) or in the same orientation as the background (Iso condition). Arrows indicate the movement of the target and the background in the opposite directions. **(C)** Example of naturalistic noise used for generating the background and the object. **(D)** Example of the random noise experiment. The target boundaries vanish in the absence of the motion signal

After arrival at the lab and a period of acclimatization to the laboratory conditions, the fish were trained to shoot at targets presented on a computer screen (VW2245-T, 21.5”, BenQ, Taiwan) situated 35 ± 2 cm above the water level (Fig. 2a). Each training session consisted of 20 trials and was conducted 2–3 times a week. First, the naïve fish were trained to shoot at a single square presented on a white background at two different locations: either in the middle of the left half of the screen or in the middle of the right half of the screen. If the fish hit the target square within 20 s after its appearance, it was rewarded with a food pellet. After 20 s the target disappeared, and the next trial started. The fish was considered ready for the experiments if it succeeded in hitting 80% of the targets in three consecutive sessions. The food reward was given manually by an experimenter immediately after the successful shot. The experimenter was sitting below the water tank level, within one-meter distance from the tank, so that the fish could not see the target and the experimenter simultaneously. Still, the experimenter was inside the fish’s field of vision, and the experimenters’ movements could have acted as unintentional cues for the fish.

### Stimuli

Stimulus images were created using Matlab. For all experiments, the stimuli consisted of a surface image shown in the background and a target square image that was cropped from a surface image. The target was placed in the middle of either the left or right half of a background image. The background image measured 26 cm x 36 cm, and the target square measured 4.5 cm x 4.5 cm.

We used three types of surface textures: sinusoidal grating, naturalistic noise patterns and random noise patterns.

### Grating

A sinusoidal grating was presented in one of 8 orientations: 0, 22.5, 45, 67.5, 90, 112.5, 135 and 157.5 degrees. The orientation of the target was either orthogonal relative to the background in the ‘cross’ condition, or parallel to the background in the ‘Iso’ condition (Fig. 2b).

### Naturalistic noise

To create the naturalistic noise texture, we used 200 natural images (https://www.kaggle.com/datasets/arnaud58/landscape-pictures). The images were transformed into the frequency domain. Then, the phase was randomized such that the total power stayed constant. Finally, using an inverse Fourier transform, we obtained an image in the spatial domain (Fig. 2c).

### Random noise

The images consisted of black and white pixels. We created 200 matrices of zeros and ones that were generated with equal probability of getting zero and one in every pixel (Fig. 2d).

The rationale for selecting these stimuli was motivated by the fact that a grating is a classical stimulus for figure-ground segregation studies in different species (Schnabel et al. 2018; Baumann et al. 1997). In addition, the two different grating conditions (Cross and Iso) generated two similar stimuli that had different levels of difficulty associated with the task. The Naturalistic noise images capture the statistical properties of the natural images. This condition was used to differentiate between a true figure-ground signal through low level processing of orientation or a phase contrast signal (Simoncelli and Olshausen 2001). Finally, the random checkerboard stimulus is an example of a pure accretion deletion signal with local information alone.

### Surface segmentation with the motion cue

In the first experiment we examined the ability of the fish to discriminate the target from the background with a motion cue. In each trial, the target moved 2 cm to the right and 2 cm to the left at a frequency of 1 Hz. The background moved at the same speed in the direction opposite to the target. After 20 s of back and forth movement, the trial ended. For the grating pattern, all the possible combinations of background angle, target angle relative to the background, and target side position were used in random order. For the Naturalistic noise and Random conditions, all 200 patterns were used, such that each pattern was novel for the fish.

### Surface segmentation without the motion cue

In the second experiment we examined the ability of the fish to discriminate the target from the background without a motion cue. We used the same patterns as in the previous experiment but removed the motion such that both the target and the background were static. The position of the target was moved 1 cm from the point where the target was cropped from the texture – the farthest position of the target relative to the background. This experiment was conducted only for the grating and natural noise textures. Random noise textures were omitted because in the static condition the target would not have been visible in this case.

### Trained stimuli vs. novel stimuli

We tested whether the performance of the fish improved with training. For this purpose, the same 4 patterns of naturalistic noise targets and backgrounds were used repeatedly in 10 consecutive sessions in random order, with 5 occurrences of each pattern in every session. We examined the success rate of the fish in response to these patterns to assess improvement in their reactions to familiar patterns as compared to their responses to the novel patterns.

### Experimental procedure

The order in which the fish completed the experiments varied for different fish. Every experimental condition consisted of 10 sessions with 20 trials per session. To keep them motivated, the sessions were conducted 3 times a week with at least one-day break between the sessions. The fish was given 20 s to respond before the trial ended and the next trial started. If during these 20 s, the fish made a shot in the direction of the target, as judged by the experimenter, it was rewarded with a food pellet. Usually, the water on the screen covered the target motion region. In addition, due to the large distance between the targets, it was easy for the experimenter to make the determination. If the fish made the shot in any other direction, the trial ended without a reward, and the next trial started. If the fish did not make a choice, the trial was removed from the accuracy calculations. Five fish out of eight fish responded to 95–100% of the trials, two fish responded to 65–85% of the trial, and one fish that completed only one type of experiment - naturalistic noise static target - responded to 48% of the trials.

### Statistical analysis

All the statistical analyses were performed in Matlab. In all statistical tests, the significance level was set at *p* = 0.01.

To verify the ability of the fish to discriminate the target from the background, we used the binomial cumulative distribution for target selection rate estimation and compared it to the chance value of 50% using a binomial test.

To test whether the responses of the fish to two types of stimuli were significantly different, for example, with and without a motion cue, we compared the binomial probabilities of the fish’s success rate.

To test if the response rates in the two conditions were equivalent, we used equivalence test of the two binomial distributions. If the difference between the probabilities was less than 0.05 on either side, the results of these two conditions were considered to be equivalent.

To evaluate the learning rate of the fish throughout the experiments, we estimated the slope coefficient of the linear model fitted to the fish’s success rate in separate sessions and tested whether it was significantly above zero. If the slope coefficient was significant, the change in success rate was considered to be significant.

## Results

We characterized the archerfish’s ability to perform segmentation of figure from background based on deletion-accretion and the discontinuity signal with and without a motion cue. For this purpose, we conducted two alternative forced choice experiments using a continuous reinforcement schedule for correct responses. The fish was presented with a stimulus of a figure over a background on a computer monitor situated above the water tank. The figure was a square moving back and forth in the opposite phase over a moving background that provided a differential motion cue with accretion-deletion. The amplitude of the figure and background motion was the same (see Methods). The fish was required to find the target and shoot at it (Fig. 2a). A shooting response directed at the figure was rewarded with a food pellet whereas shooting elsewhere was not rewarded and counted as an incorrect response. An incorrect response resulted in the termination of the trial and the initiation of the next one. To neutralize the effect of position bias in the fish’s responses, the figure was presented at random in two locations on the screen.

We tested archerfish segmentation behavior using four classes of stimuli: (1) A cross grating stimulus, where the figure consisted of a grating and the ground consisted of an orthogonal grating (Cross condition, Fig. 2b). (2) A grating stimulus where the figure consisted of a grating, and the ground consisted of a grating in the same orientation, but with an offset in phase (Iso condition, Fig. 2b). The background orientation was selected at random from 8 angle options (Background angles, Fig. 2b). (3) Naturalistic stimuli, where both the figure and the ground consisted of naturalistic noise patterns (Naturalistic condition, Fig. 2c, see methods). (4) A random checkerboard noise where each pixel was selected randomly from a binary distribution (Random condition, Fig. 2d).

### Archerfish can segment objects defined by opposing motion

**Fig. 3:**
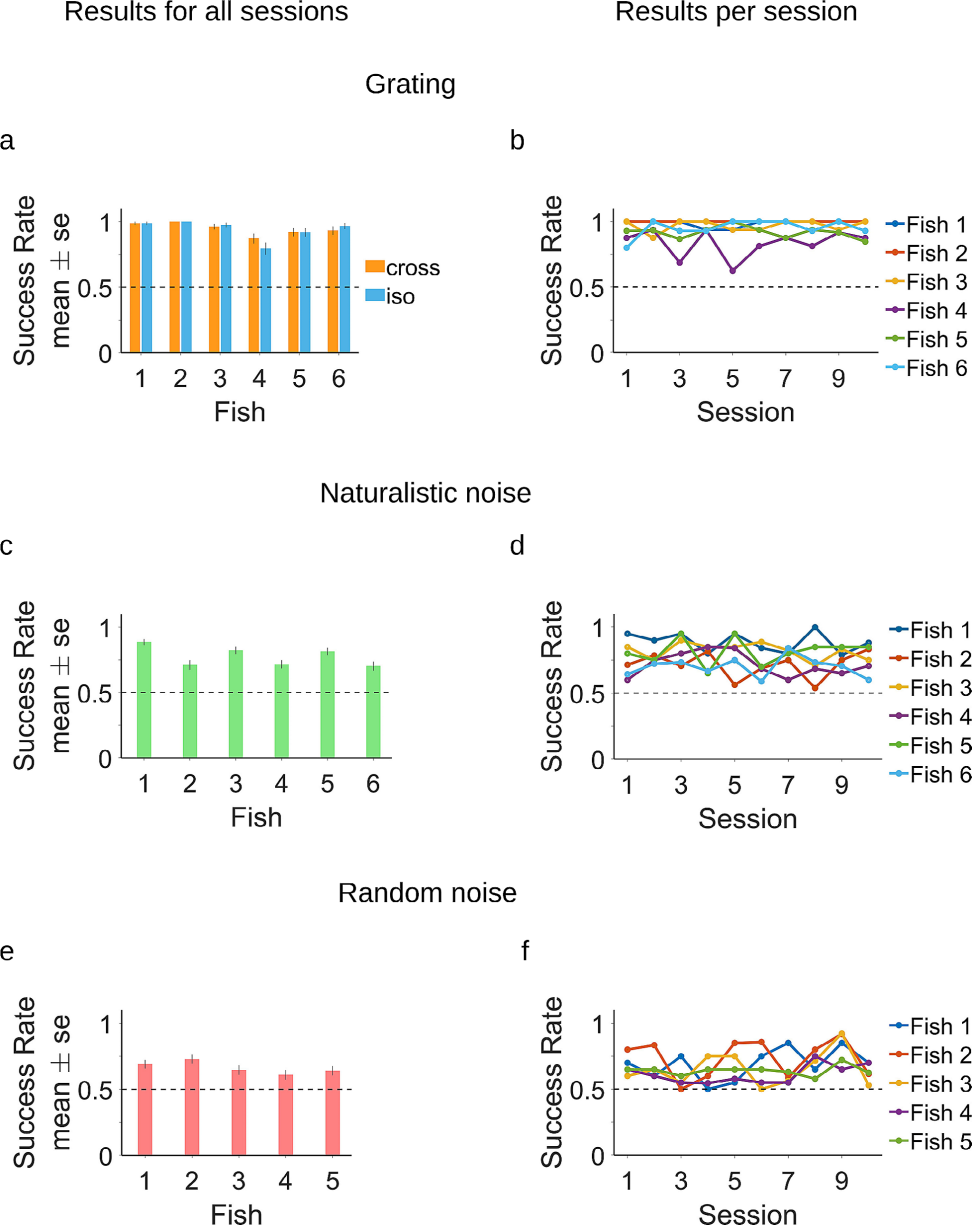
Archerfish can perform figure-ground segmentation with a motion signal. **(A)**. Average success rate of the fish on the segmentation task in the grating Iso and Cross conditions. **(B)** Average success rate of the fish per session. The detection of the target in the Cross and Iso conditions achieved almost perfect performance. **(C)** Success rate of the fish in the segmentation task in the naturalistic noise condition. **(D)** Success rate of the fish per session. **(E)** Random noise condition. **(F)** Success rate of the fish per session. In the naturalistic noise and random noise conditions, the fish performed significantly above chance but lower than perfect performance. The fish’s success rate did not increase over sessions indicating that there was no learning in the segmentation process

The results showed that the archerfish successfully performed the task based on the motion signal. The archerfish performed well from the first session of the grating stimulus presentation and maintained their nearly perfect performance thereafter (Fig. 3a, b). This nearly perfect performance was observed in both the Cross and the Iso condition in all fish (binomial test, highest p-value – 2*10^− 8^).

In addition to the grating experiment, the fish were presented with the Naturalistic noise stimulus, where no local orientation signal was present and thus could not be used for segmenting the figure from the ground. Again, the archerfish were able to perform the task at a success rate above chance (binomial test, highest p-value – 8.5*10^− 8^), although below their almost perfect performance in the grating experiment (Fig. 3c, d).

Finally, the fish performed surface segmentation in the extreme case of the Random condition. In this case, the figure-ground segmentation relied on the accretion-deletion signal alone. The fish were able to perform the task above chance level (binomial test, highest p-value – 0.001; Fig. 3e, f). For four out of five fish, the success rate was lower than in the grating or natural noise (comparison of two binomial distributions, highest p-value – 1.4*10^− 4^); for one fish, the result of the statistical test was not conclusive (p-value – 0.14; Fig. 3a, c, e).

Thus overall, archerfish can segment surfaces based on the opposing signal between figure and ground. This was observed for a wide variety of stimuli but with decreasing levels of success as the task became harder.

### Archerfish segmentation in natural noise relies on the motion signal alone

Next, we examined whether the archerfish used the motion signal or some other local contrast for segmentation. For this purpose, we tested the ability of the fish to detect the same stimuli when the target was not moving, to test how critical the motion signal is in three conditions: cross grating, iso grating, and naturalistic noise. Five fish started with the moving target condition before moving to the static target, three fish did the static condition first.

**Fig. 4:**
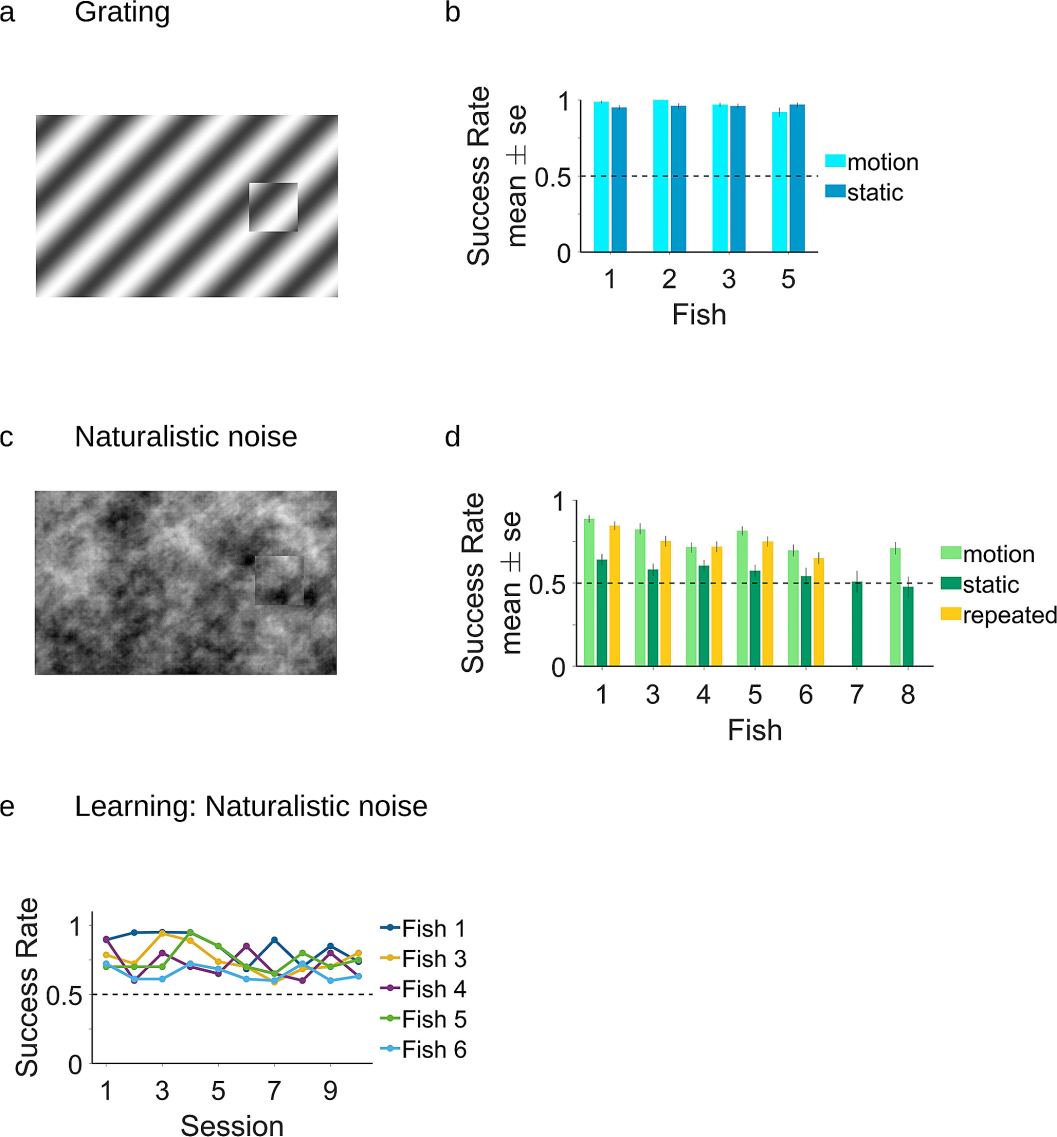
Comparison between motion cue and static cues for different stimulus types and repeated stimuli. **(A)** Example of the grating stimulus. **(B)** In the grating stimulus condition, the fish detects the target and performs the segmentation process in both conditions, with and without the motion cue. **(C)** Example of the naturalistic noise stimulus. **(D)** In the naturalistic noise case, the fish can perform segmentation using the motion cue but fail to do so in the static condition (light green vs. dark green). There was no significant difference in fish performance in the case of novel vs. repeated stimuli (light green vs. yellow) **(E)** Success rate of the fish in the repeated naturalistic noise condition per session. The fish did not show any clear learning in terms of improvement over sessions

We found that the fish were able to detect the target in both grating cases (Fig. 4a, b; binomial test, highest p-value – 3.3*10^− 20^). This is not surprising since in both the Cross and Iso cases, the single static frame still has an edge contrast as a result of orientation or phase differences. Thus, the image contained clear figure edges that could be detected. Fish performance with the static target was comparable to the performance with the moving target (equivalence test with 5% accepted difference, highest p-value – 2*10^− 7^).

In contrast, the fish struggled to detect the object in the case of static naturalistic noise. Five out of seven fish performed near chance level (lowest p-value – 0.04) and did not improve over sessions (Fig. 4c, d, motion and static). Two out of these fish started with naturalistic noise and had chance level performance, similar to the fish that started with other types of stimuli within the naturalistic noise condition.

Only two fish succeeded in detecting the target (highest p-value – 0.001), but the success rate was significantly lower than for the moving target. This was expected since the discontinuity cue from the boundary is much weaker in the case of naturalistic noise than in the grating condition (see, for example, Fig. 4a, c). Hence, motion is a critical component of the fish’s ability to perform figure-ground segmentation.

Overall, these experiments confirm that the archerfish used the motion signal to perform figure-ground segmentation in the natural noise case. The results for the grating experiment indicate that the fish used different strategies in the naturalistic and grating conditions. It is important to note that the random condition was not tested since, in this case, there is no signal of the target position. We therefore omitted this condition from the experiment.

### Response to repeated stimuli shows that archerfish do not learn the task

We tested the ability of the archerfish to perform object segmentation on a naturalistic noise background using either novel or repeated stimuli. Three out of five fish that completed both experiments, first performed the novel stimuli type, then the repeated type; for two fish, the order was reversed. In the novel stimuli experiment, 200 different non-repeated stimuli were used. We looked at the average response of the fish and also at the change in success rate over the course of the 10 sessions of the experiment. We observed variability in the fish’s responses across different sessions that reached 30% in some fish, but no improvement over time. The average success rate in the first sessions of the experiments was similar to the success rate in the last sessions (comparison between two binomial distributions of the two first and the two last sessions for all fish, lowest p-value – 0.22).

We also assessed whether the fish would improve their performance if we trained them on the same repeated backgrounds. We used 4 different backgrounds and presented them to the fish in 10 sessions, with 5 repetitions of each background per session. We observed no improvement in any of the five fish that completed the experiment (Fig. 4e). To calculate the learning rate and test its significance, we fitted a linear model to the success rate of each fish in every session. None of the slope coefficients of the fitted linear models was significant (lowest p-value – 0.25). This is an indication that learning, if it exists, must be very weak. When we compared the average success rate in fish’s responses to novel vs. repeated backgrounds (Fig. 4d, motion and repeated), results were similar: for all fish, the success rates were not statistically different (lowest p-value – 0.05).

### Comparing segmentation behavior in archerfish, rodents, and primates

**Fig. 5:**
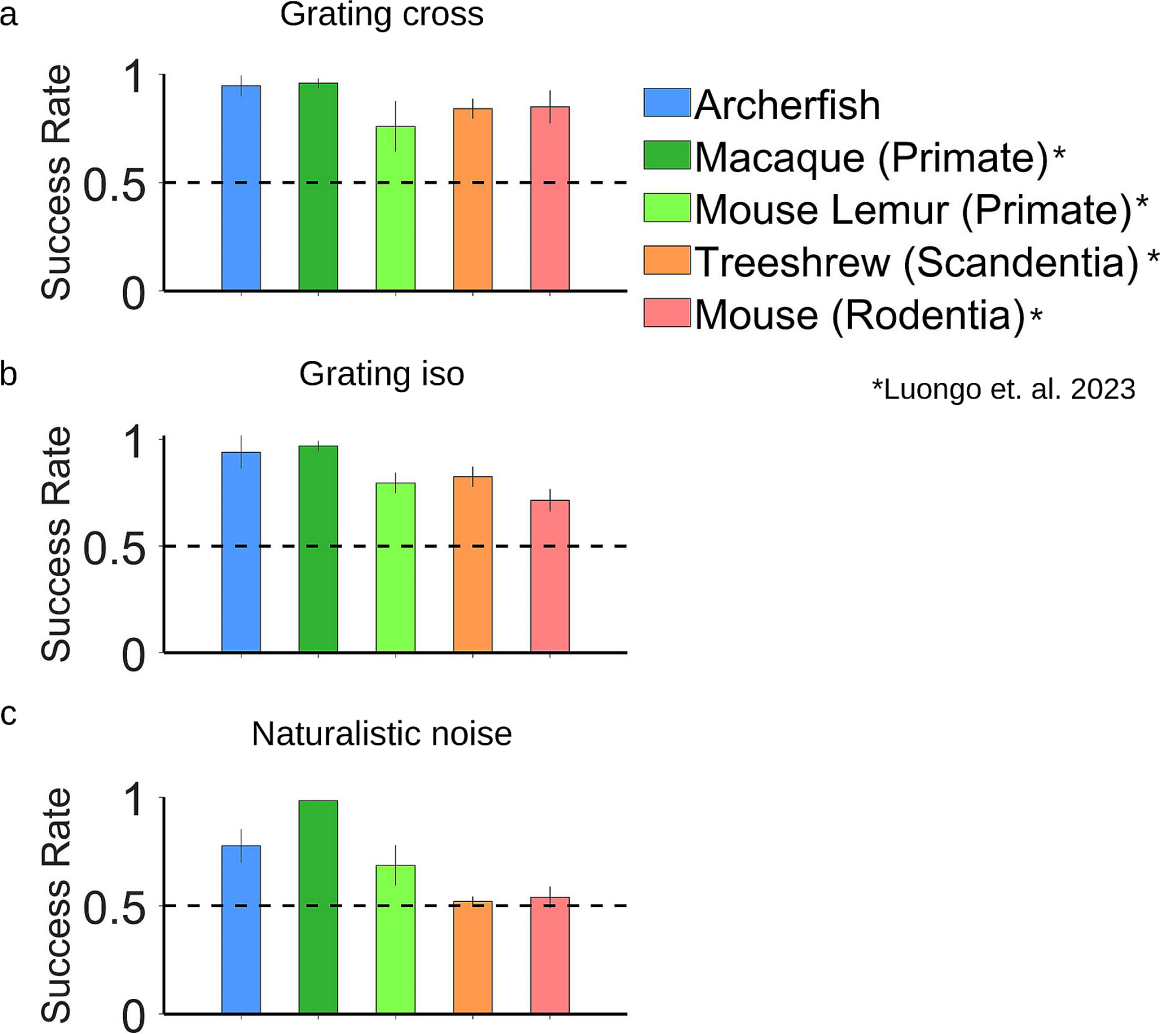
Comparison of archerfish to other species. Comparison of the ability of the archerfish on different tasks indicates that the archerfish is comparable to primates since it was successful on both the grating and naturalistic noise conditions using the motion cue

In a recent study (Luongo et al. 2023), the segmentation behavior of four different mammalian species was studied in two primate species – the macaque and mouse lemur – mice of the *Rodentia* order and treeshrews of the *Scandentia* order. This provided an opportunity to compare the archerfish segmentation behavior with these species since the experimental procedures were identical. Figure 5 presents a comparison of the success rate of these different species. In the grating experiments, the archerfish, like the other species, was able to perform the task in both the Iso and Cross conditions. The archerfish and the primates exhibited slightly better performance (Fig. 5a, b). In the naturalistic noise condition, a different result emerged. The archerfish and primates could identify the target defined by an accretion-deletion signal whereas rodents performed at chance level (Fig. 5c). Hence the archerfish figure- ground segmentation appears to be comparable to primates. Specifically, the archerfish performance can be considered to be situated between the macaque and the mouse lemur.

## Discussion

We studied the ability of archerfish to perform figure-ground segmentation based on different cues: accretion-deletion, discontinuity and a combination of both. In the case of accretion-deletion, the segmentation process relies on the accretion-deletion of pixels before and after the moving figure. We used grating, naturalistic noise, and random noise as targets moving counterphase with the background.

We found that the archerfish succeeded in delineating the target from the background in all types of stimuli when a motion cue was present. The fish were also able to generalize from the moving targets to the static targets, since they performed the grating task at a high success rate from the very first session. The archerfish needed the motion signal in the case of the naturalistic noise because the fish failed to detect the target in the static case.

This study draws on Luongo et al. 2023, who examined the ability of several mammalian species across three lineages to perform a figure-ground segmentation task. Their work underscored the importance of a comparative study of the mechanism of figure-ground segmentation. They found diversity in performance across mammalian lineages, which is indicative of the inherent differences and difficulties in the processing of objects across species. Specifically, they found that mice (order *Rodentia*) and treeshrews (order *Scandentia*) were less able to exploit a pure motion signal as compared to primates where the mice could not perform the naturalistic noise condition based on the motion signal. We used the same stimuli to enable direct comparison across taxa.

We found that archerfish are comparable to the primates in their capacity to use a motion signal combined with discontinuity to perform figure-ground segmentation (Fig. 5). This was true in the case of their success rate under different conditions with the moving target and their inability to perform the task when the target was static.

This finding is important since it indicates that the capacity to use a motion signal to segment figures from the background has evolved independently across taxa (Nilsson 2009). As fish and mammals diverged about 450 MY ago, the ability to perform segmentation based on a motion signal has probably evolved several times whenever this ability was needed. An additional option is that this ability is so fundamental that it predates the split and is ancestral to all vertebrates.

Previous literature on figure ground segmentation in fish has only examined conditions where the visual cues consisted of intensity or orientation contrast in redtail splitfins (Sovrano and Bisazza 2009) and in archerfish (Mokeichev et al. 2010). These works reported that fish can perceive subjective, or illusionary, contours when these contours lack a physical counterpart in terms of luminance contrast gradients. These contours can be based on either continuity with nearby real contours or changes in texture that define the contour. These studies differ from the work reported here since the segmentation process did not use motion as a cue. The present study is thus the first to focus on motion as the single unambiguous cue for figure-ground segmentation in fish.

In the archerfish, studies on object recognition generally deal with cases where the object could be easily segmented from the ground based on discontinuity of intensity. This was observed in both the case of object recognition of naturalistic objects (Volotsky et al. 2022) and in the case of human face recognition (Newport et al. 2018). In addition, in previous work, the targets did not move, although it is well-known that motion is a salient visual cue for the archerfish visual system (Ben-Tov et al. 2015; Reichenthal et al. 2019).

Another lineage that has received considerable attention in the literature on figure-ground segmentation is birds, especially pigeons (Lazareva et al. 2006; Clark et al. 2022). Studies on birds suggest they may attend to the figural region rather than use local properties when performing figure-ground segmentation. It was suggested that specific regions in the brain might be responsible for figure-ground segmentation (Scully et al. 2014). Recent work on the barn owl tested its ability to detect a figure using the animal’s self-motion and found that information about self-motion can facilitate orientation-based figure-ground segmentation (Dutta et al. 2020). The present study is complementary to these works since we explored segmentation in another taxon.

The study of figure-ground segmentation across species raises the question, what is the neural basis of this visual processing capability? As knowledge about the function of single cells in the early visual system is still lacking in some species and exists in others (Luongo et al. 2023; Reichenthal et al. 2018; Self et al. 2013; Baumann et al. 1997) there is a need for future work to address this question in full.

## Conclusion

We examined the ability of archerfish to segment figure from ground based on motion cues. Indeed, the archerfish were able to use the motion signal, however, depending on the stimuli, the fish could use additional orientation signal for segmentation. Future studies should explore whether and how this visual segmentation process is carried out by the neural circuitry responsible for object recognition in the archerfish.

## Data Availability

Data will be available after publication at github.
